# Supplementary material for: Concurrent Reactive Oxygen Species Generation and Aneuploidy Induction Contribute to Thymoquinone Anticancer Activity
Source: Molecules. 2021 Aug 25;26(17):5136. doi: 10.3390/molecules26175136 (PMC8433634; doi:10.3390/molecules26175136)
Supplement: Supplementary file 1 [file molecules-26-05136-s001.zip › molecules-1323312-supplementary.pdf]

Concurrent Reactive Oxygen Species-Generation and Aneuploidy-Induction Contribute to Thymoquinone Anticancer Activity.

Mohammed Al-Hayali [1], Aimie Garces [2], Michael Stocks [2], Hilary Collins [2] and Tracey D. Bradshaw [2,*]

[1] Al-Mosul General Hospital, P.O.Box:11104-Mosul University post office, Iraq, Postcode: 41002
[2] School of Pharmacy, Biodiscovery Institute, University of Nottingham, University Park, Nottingham, NG7 2RD, UK; aimie.garces@nottingham.ac.uk (A.G.); michael.stocks@nottingham.ac.uk (M.S.); hilary.collins@nottingham.ac.uk (H.C.)
* Correspondence: mohammedalhayali@gmail.com (M.A.); bradshaw@nottingham.ac.uk (T.B.)

Supplementary Information

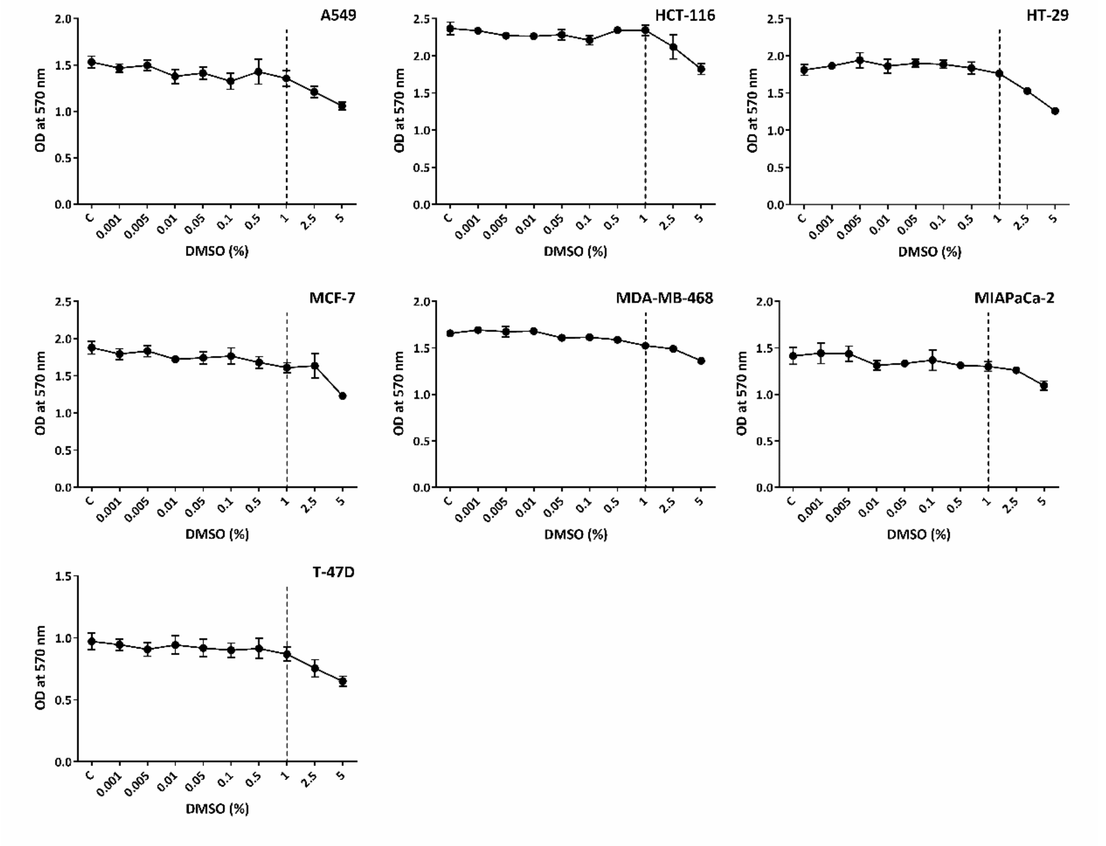

Figure S1. Line graphs show DMSO effects on the growth of A549, HCT-116, HT-29, MCF-7, MDA-MB-468, MIAPaCa-2 and T-47D. Each representative graph shows one independent MTT trial. DMSO (%) is plotted against optical density. Cells were cultured for 24 h in 96-well plates (3×10^3 cells/180 μL medium/ well) then treated with DMSO alone for 72h. Dashed line shows DMSO 1% treatment group (which corresponds to 100 μM TQ concentration) at which no significant growth inhibition was seen with DMSO alone in all cell lines. No. trials ≥3; n=4 per independent experiment.

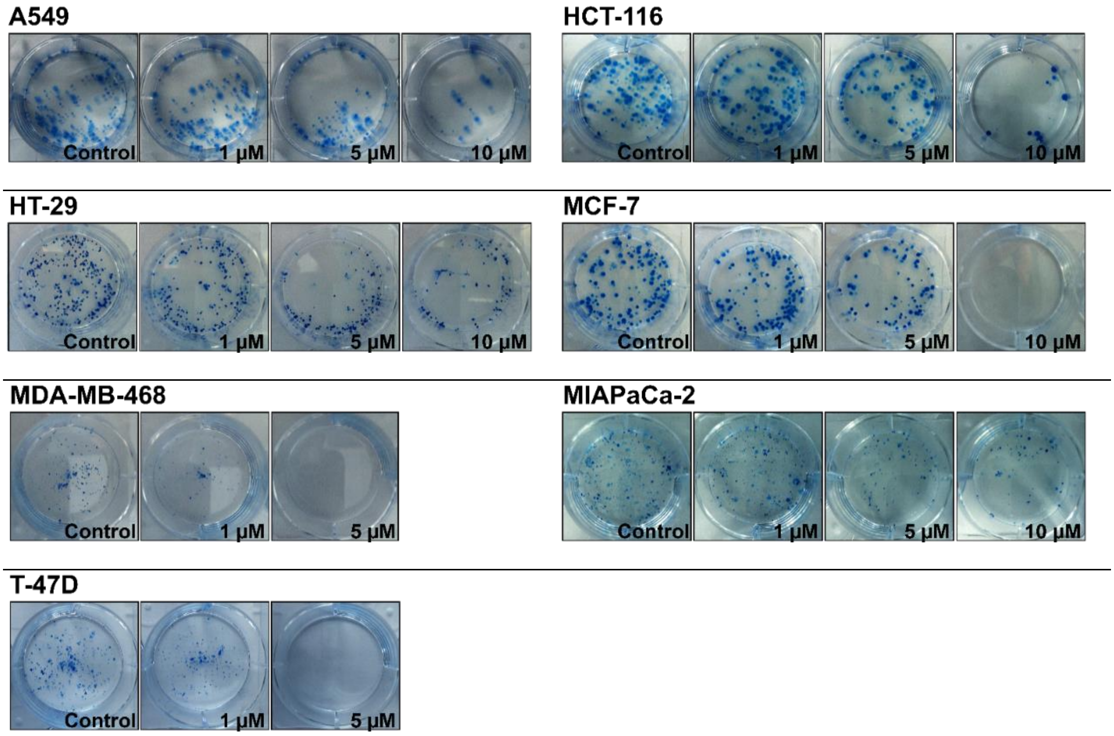

Figure S2. Representative photos of TQ`s inhibition of A549, HCT 116, HT-29, MCF-7, MDA-MB-468, MIAPaCa-2 and T-47D colonies. Mean±SD bars show mean survival fraction as % of control. Cells were seeded, treated with TQ (24h) then medias replaced with fresh ones. When colonies contained ≥50cells, colonies were fixed, stained and counted. Plating efficiencies ranged between 20% and 35%. Assays repeated 3times (n=2).

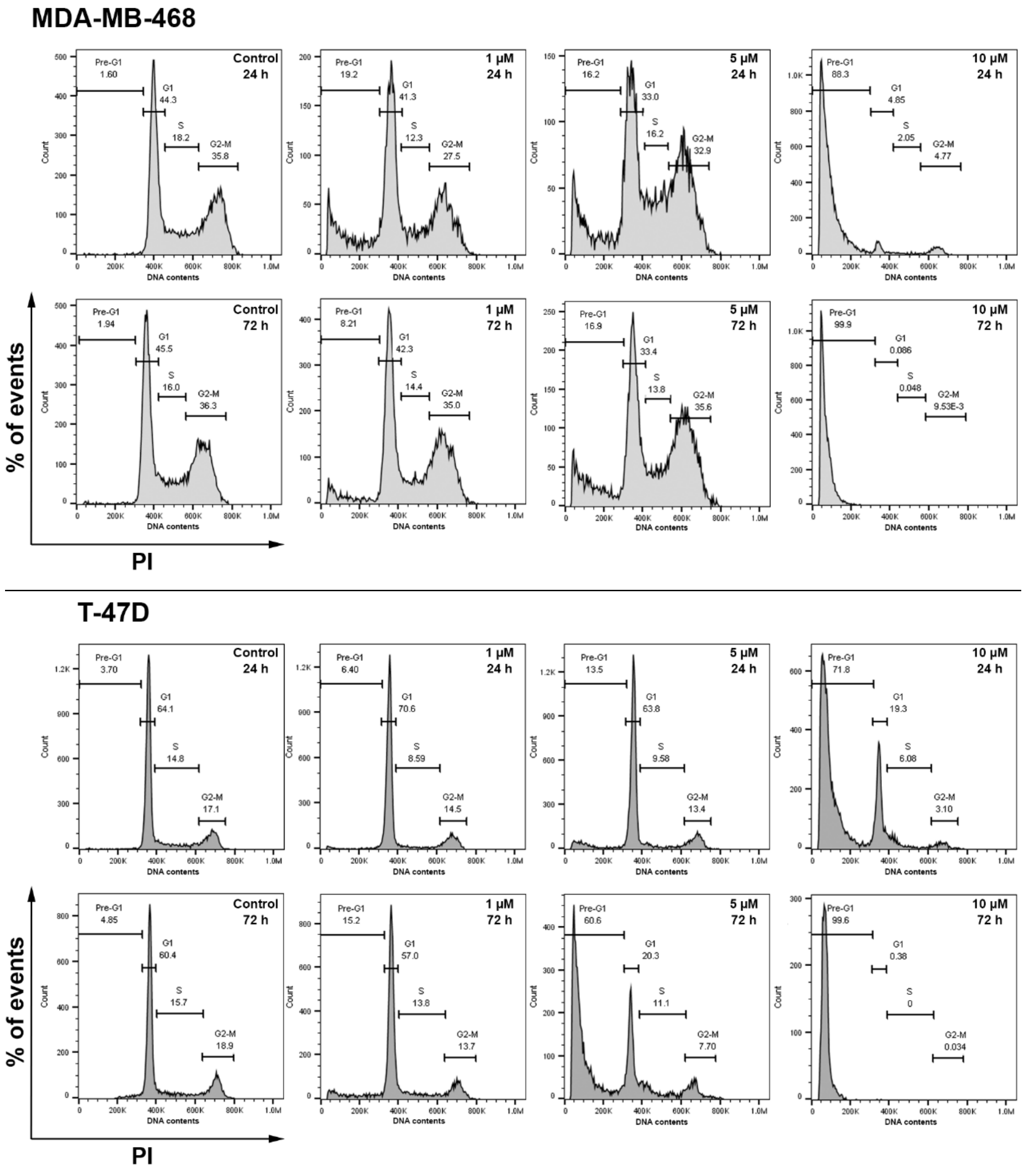

Figure S3. Representative cell-cycle histograms from one independent trial showing % events distribution of MDA-MB-468 and T-47D following 24, 72 h (1, 5, 10 µM) TQ exposures. TQ induced significant concentration- and time-dependent increase in pre-G1(<2N) with decreased other cell-cycle phases. Cells were treated; then stained with PI and at least 20,000 events/sample were measured. Assays repeated 3 times (n=2).

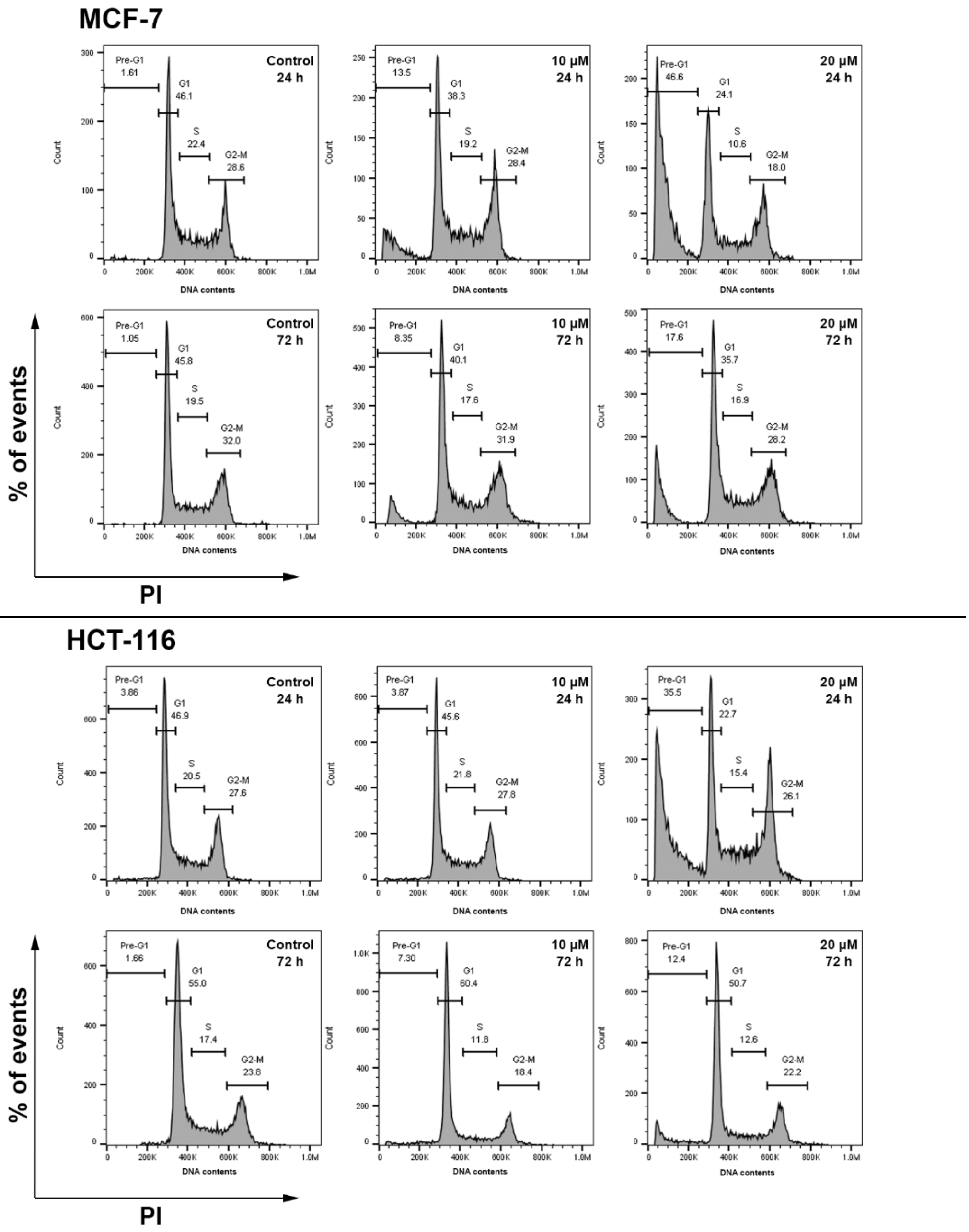

Figure S4. Representative cell-cycle histograms from one independent trial showing % events distribution of MCF-7 and HCT-116 following 24, 72 h (10, 20 µM) TQ exposures. After 24 h, TQ induced significant concentration-dependent increase pre-G1(<2N). After 72 h, less pre-G1 events were seen. Cells were treated; then stained with PI and at least 20,000 events/sample were measured. Assays repeated 3 times (n=2).

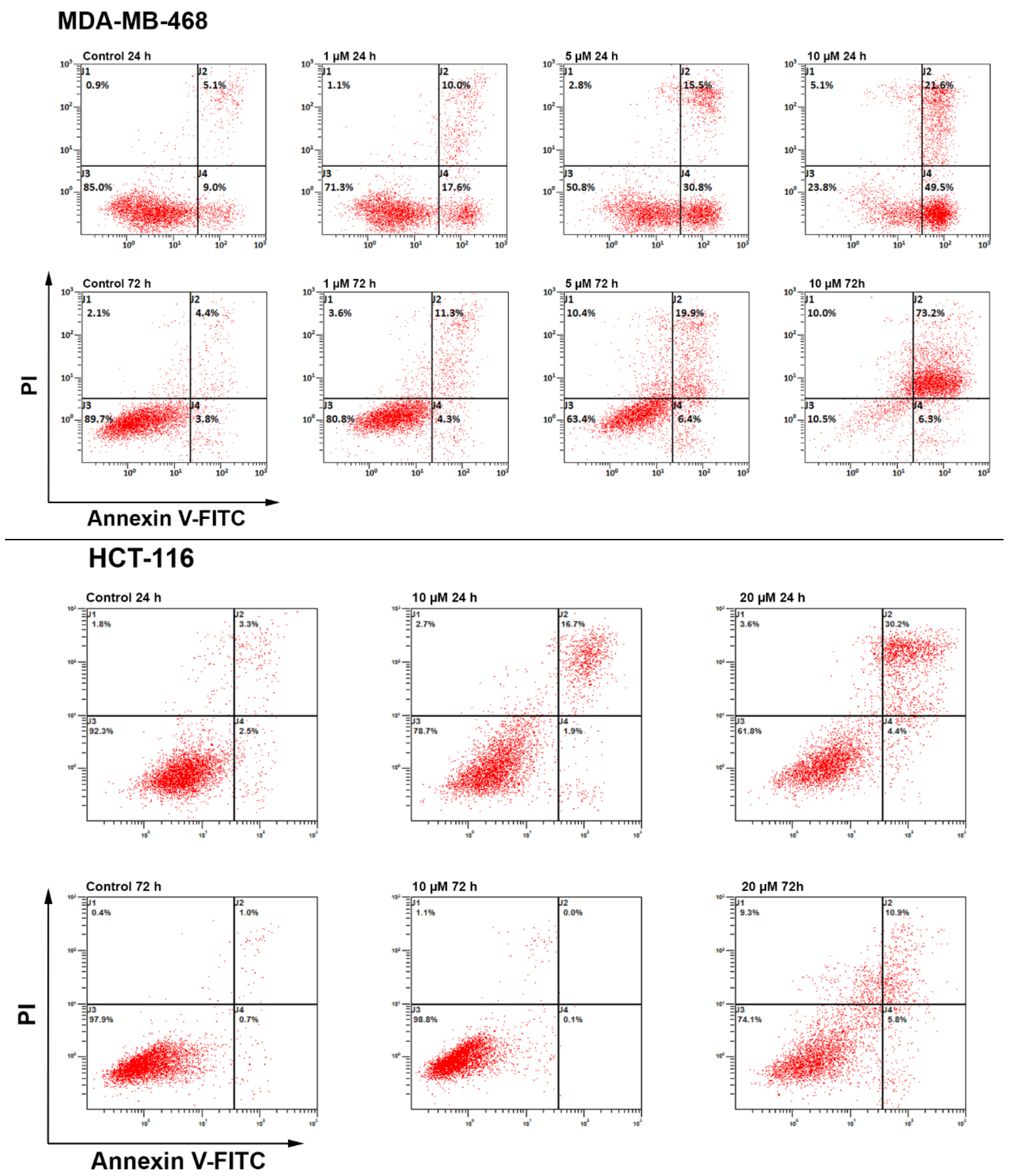

Figure S5. Representative dot-plots from one independent trial showing annexin V/PI results of MDA-MB-468 and HCT-116 cells treated with TQ for 24, 72 h. MDA-MB-468 treated with (1,5,10 µM) and HCT-116 treated with (10,20 µM). TQ caused significant concentration-dependent increase in apoptotic events. In MDA-MB-468 and after 24 h, TQ induced early-apoptotic (A+/PI-) more than late-apoptotic (A+/PI+) events; after 72 h, majority of apoptotic cells were shifted to late-apoptosis quadrate (A+/PI+). In HCT-116 and after 24 h, TQ also caused significant increase in apoptotic events and majority of them were late-apoptotic (A+/PI+) but after 72 h, HCT-116 recovered from TQ-induced apoptotic effects. Samples were stained with annexin V/PI and at least 10,000 events were detected. The percentage of apoptotic events was equal to sum of cells undergoing early-apoptosis (A+/PI-) plus late-apoptosis (A+/PI+). Assays repeated 3 times (n=2).

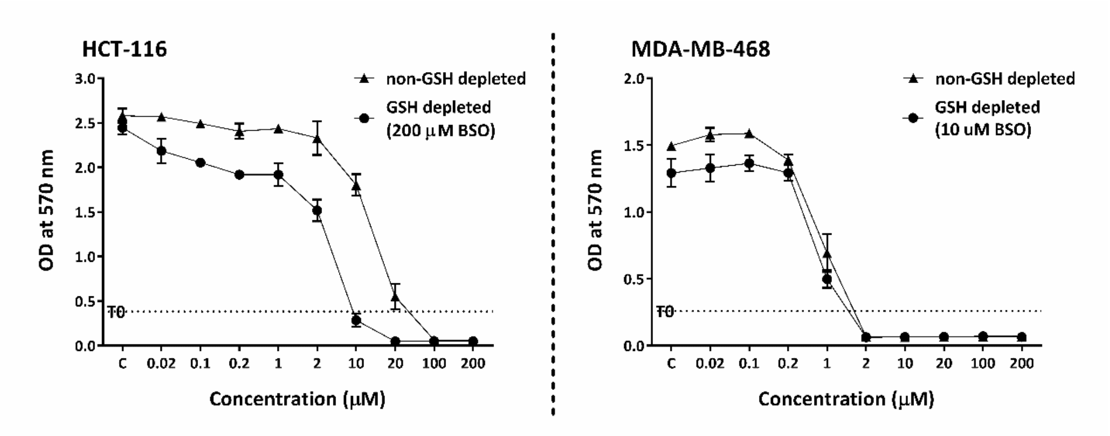

Figure S6. Line graphs show growth inhibitory effects of TQ in MDA-MB-468 and HCT-116 either GSH -continuously depleted or -not depleted by BSO. Each representative graph shows one independent MTT trial. Cells were seeded in 96-well plates (3×10^3 cells/well) and treated with BSO for 72 h. For GSH-depleted cells, BSO was introduced for 24 h after which TQ was added to both GSH depleted and not depleted cells for 72 h. Assays repeated 3 times (n=4).

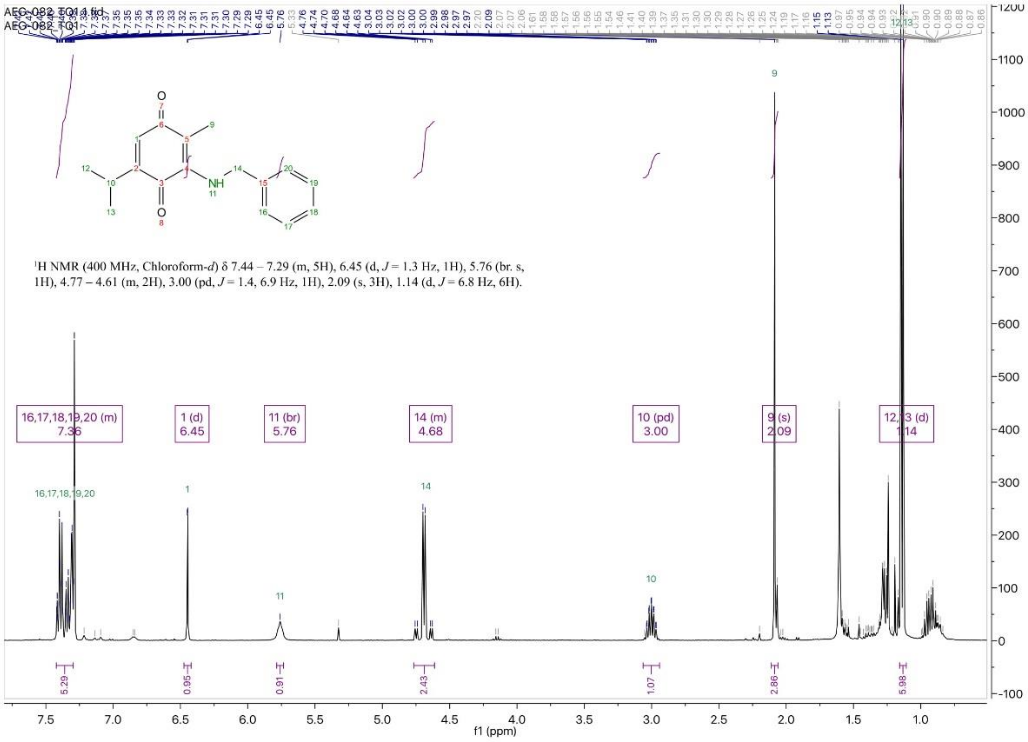

Figure S7. ^{1}H NMR spectrum (1-dimensional) of TQ1 plotted as signal intensity (vertical axis) vs. chemical shift in ppm (horizontal axis). Signals from spectrum have been assigned hydrogen atom groups (a through j) from the structure shown at upper left.

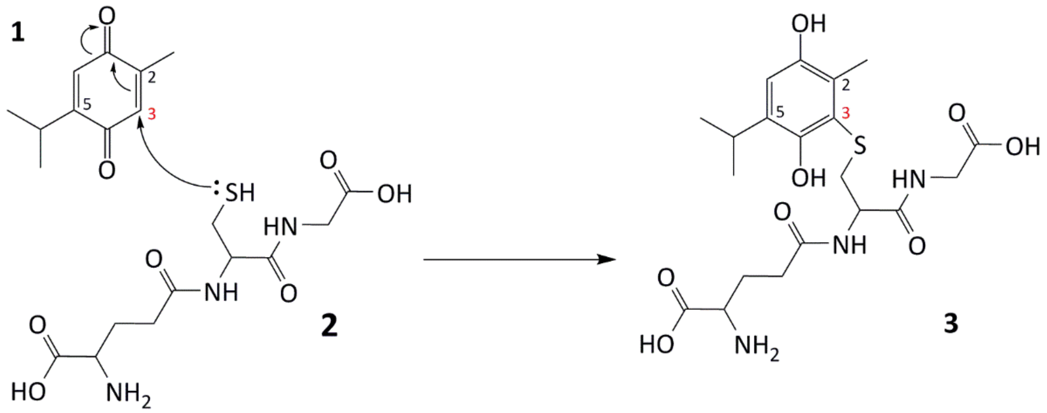

Figure S8. Michael Addition reaction of GSH (**2**) and TQ (**1**) with SH group attacking carbon 3 of TQ leading to formation of glutathionyldihydro-TQ (**3**).
